# Effects of feeding *Saccharomyces cerevisiae* fermentation postbiotic on the fecal microbial community of Holstein dairy calves

**DOI:** 10.1186/s42523-023-00234-y

**Published:** 2023-02-19

**Authors:** Ruth Eunice Centeno-Martinez, Wenxuan Dong, Rebecca N. Klopp, Ilkyu Yoon, Jacquelyn P. Boerman, Timothy A. Johnson

**Affiliations:** 1 Department of Animal Science, Purdue University, 270 S Russell St., West Lafayette, IN USA; 2 Diamond V, Cedar Rapids, IA USA

**Keywords:** Calf, Fecal microbiome, Maturation, *Saccharomyces cerevisiae* fermentation product

## Abstract

**Background:**

The livestock industry is striving to identify antibiotic alternatives to reduce the need to use antibiotics. Postbiotics, such as *Saccharomyces cerevisiae* fermentation product (SCFP), have been studied and proposed as potential non-antibiotic growth promoters due to their effects on animal growth and the rumen microbiome; however, little is known of their effects on the hind-gut microbiome during the early life of calves. The objective of this study was to measure the effect of in-feed SCFP on the fecal microbiome of Holstein bull calves through 4 months of age. Calves (n = 60) were separated into two treatments: CON (no SCFP added) or SCFP (SmartCare®, Diamond V, Cedar Rapids, IA, in milk replacer and NutriTek®, Diamond V, Cedar Rapids, IA, incorporated into feed), and were blocked by body weight and serum total protein. Fecal samples were collected on d 0, 28, 56, 84, and 112 of the study to characterize the fecal microbiome community. Data were analyzed as a completely randomized block design with repeated measures when applicable. A random-forest regression method was implemented to more fully understand community succession in the calf fecal microbiome of the two treatment groups.

**Results:**

Richness and evenness of the fecal microbiota increased over time (*P* < 0.001), and SCFP calves tended to increase the evenness of the community (*P* = 0.06). Based on random-forest regression, calf age as predicted by microbiome composition was significantly correlated with the calf physiological age (R^2^ = 0.927, *P* < 1 × 10^−15^). Twenty-two “age-discriminatory” ASVs (amplicon sequence variants) were identified in the fecal microbiome that were shared between the two treatment groups. Of these, 6 ASVs (*Dorea-*ASV308, *Lachnospiraceae-*ASV288, *Oscillospira*-ASV311, *Roseburia*-ASV228, *Ruminococcaceae-*ASV89 and *Ruminoccocaceae*-ASV13) in the SCFP group reached their highest abundance in the third month, but they reached their highest abundance in the fourth month in the CON group. All other shared ASVs reached their highest abundance at the same timepoint in both treatment groups.

**Conclusions:**

Supplementation of SCFP altered the abundance dynamics of age discriminatory ASVs, suggesting a faster maturation of some members of the fecal microbiota in SCFP calves compared to CON calves. These results demonstrate the value of analyzing microbial community succession as a continuous variable to identify the effects of a dietary treatment.

**Supplementary Information:**

The online version contains supplementary material available at 10.1186/s42523-023-00234-y.

## Background

Dairy calf feeding systems seek to provide pre-weaned calves with dietary nutrition to promote immediate growth and health, as well as future milk production. An important factor in the animal nutrition is the gut microbiome. The gut microbiota has been identified to be important for the development of the intestinal epithelium, mucosal layer, and immune cell repertoire [1]. Additionally, studies had identified a potential link between the small intestine microbiome with calf immune function, health and growth [2, 3]. As an example, a study identified that the prevalence of *Faecalibacterium* spp. in the fecal microbiota of neonatal calves in the first week of life was associated with higher weight gain and less diarrhea [4]. Therefore, it is important to maintain calf gut health to minimize susceptibility to enteric infections and to improve the animal health and growth. Additionally, the increasing prevalence of antibiotic-resistant bacteria in both animals and humans has led to the need for alternatives to antibiotics that can still promote the same positive calf health and growth benefits that antibiotics currently provide.

Postbiotics can be defined as a mixture of intermediate and end products from microbial fermentation that can contribute to observed health benefits, and are currently being explored as non-antibiotic growth promoters [5]. One example of a postbiotic is *Saccharomyces cerevisiae* fermentation product (SCFP), which is produced during the anaerobic fermentation of *Saccharomyces cerevisiae* and provides a complex mixture of metabolites, including lysed cell components, amino acids, lipids, volatile fatty acids, and B vitamins [6]. In the dairy industry, SCFP has supplemented the calf diet to improve feed intake, growth, health and rumen development [7–9]. SCFP improves rumen fermentation, which can be measured by VFA concentration, blood glucose levels, ruminal pH, concentration of anaerobic and cellulolytic bacteria, and papillae length [10–12]. In cows challenged with sub-acute rumen acidosis (SARA), a health disorder in which a the rumen suffers a reversible reduction of the pH below 5.6 and 5.8 for a prolonged period [13], supplementation of 14 g·d^−1^ of SCFP did not cause a change in the dry matter digestibility and total tract digestibility of crude protein and phosphorus, but supplementation of 38 g·d^−1^ of SCFP resulted in increased digestibility of neutral detergent fiber (NDF), which includes hemicellulose, cellulose and lignin compounds [14]. In vitro studies using rumen activity modifier model (RAMM) have shown that supplementation of SCFPs increases the abundance of fibrolytic and lactate utilizing organisms compared to control [13]. A follow up metabolic challenge study in mid-lactation dairy cows showed that SCFP supplementation attenuated the negative effects of subacute ruminal acidosis (SARA) such as reduction of richness and evenness of the rumen microbiota, reduction of beneficial fibrolytic bacteria and protozoa and increase the abundance of pathogenic bacteria [13]. Only recently, in a companion article to the current study, it was found that supplementing the calf diet with SCFP improved average daily gain (ADG), body weight (BW), and hip width (HW) in comparison to the control group. In addition, animals that received SCFP had a reduction in respiratory disease incidence post-weaning [15]. We hypothesized that due to the other health benefits observed, supplementing calves with SCFP, would increase alpha diversity, alter community structure and increase the rate of community succession of the fecal microbiota when compared to the CON. Therefore, the objective of this study was to further evaluate the effects of SCFP, when supplemented in milk replacer and calf starter, on the dairy fecal microbiome composition before and after weaning and community succession during the first 4 months after birth.

## Results

**Fig. 1:**
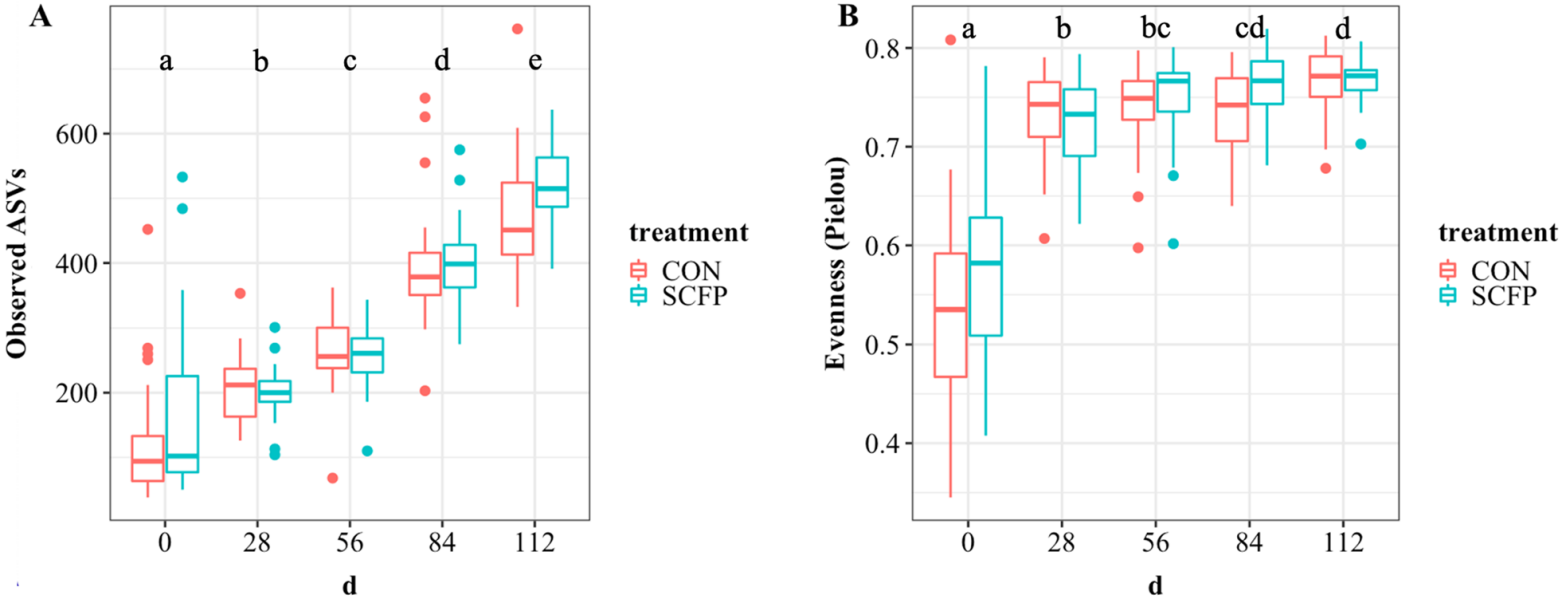
Calf fecal alpha diversity measured by observed ASVs (**A**) and Pielou Index (evenness) (**B**) was significantly affected by time (0, 28, 56, 84, and 112 d). Letters (a–e) indicate significant differences (*P* < 0.05) between each time point but not between treatments

A total of 7529 ASVs were observed in the study. However, to calculate the alpha and beta diversity of the fecal microbiome, samples were rarified to 19,681 reads per samples, resulting in 6212 ASVs remaining for use in alpha and beta diversity analysis. The microbial richness predicted by the observed ASVs index was not affected by diet treatment (*P* = 0.17). On the other hand, observed ASVs increased with time (*P* < 0.0001; Fig. 1A), with every day (0, 28, 56, 84, and 112 d) being different from the others, regardless of the dietary treatment. Additionally, Holstein bull calves used in this study were received in two separate batches (30 calves per batch, with 15 calves per treatment per batch). We found that batch 2 had a higher number of observed ASVs compared to batch 1 (*P* < 0.001). The microbial community evenness measured with the Pielou Index (Fig. 1B) increased over time (*P* < 0.001), and evenness was increased in batch 2 compared to batch 1 (*P* = 0.04). The evenness of the community tended to be greater in SCFP calves compared to CON (*P* = 0.06).

**Fig. 2:**
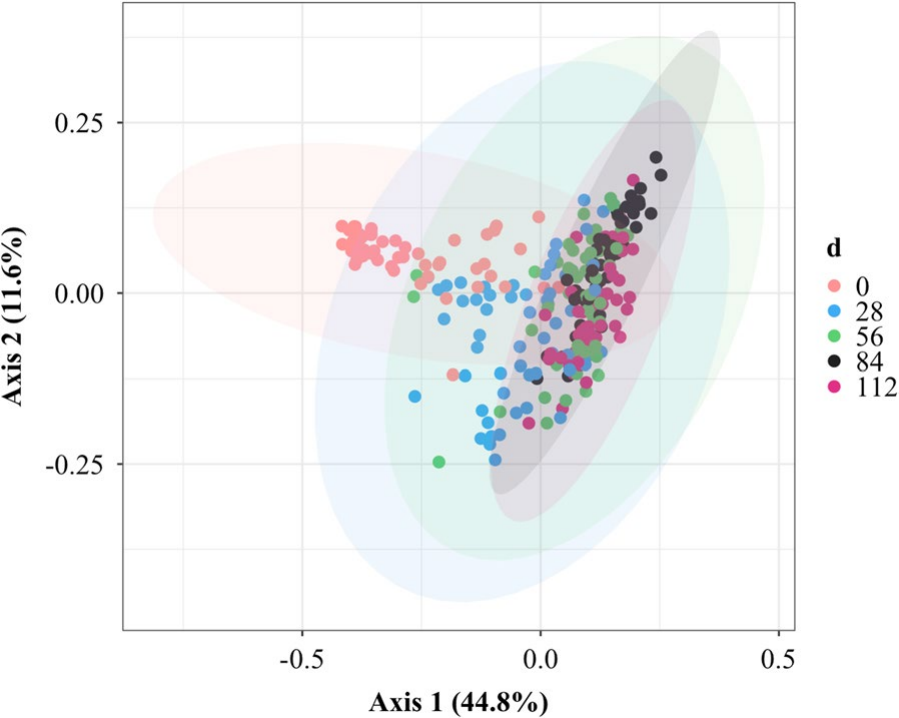
Principal coordinates analysis plot illustrating the beta diversity of the calf fecal microbiota at each time point (0, 28, 56, 84, and 112 d) estimated by the Weighted UniFrac distances

Beta diversity of the calf fecal microbial community was not affected by diet treatment on days 28, 56, and 84, but on day 112 the community composition was affected by diet treatment (*P* = 0.03). Dispersion of the samples from the centroids of each treatment group was not different on day 112 (distances CON: 0.1263, SCFP: 0.1259, *P* = 0.96). Batch affected the calf fecal microbial community at the time points 0, 28, 56, 84, and 112 d (all *P* < 0.003). The dispersion of the samples from batch group centroids was only different on 56 (distances B1: 0.1283 and B2: 0.1774, *P* = 0.006) and 112 d (distance B1: 0.1025 and B2: 0.1448, *P* = 0.001). Block affected the fecal microbial community on 84 and 112 d (both *P* < 0.02). However, based on the pairwise comparisons between the three different batches on day 84 and 112, no pairwise difference was observed (*P* > 0.05). Additionally, the dispersion of the samples on 112 d were different between the blocks (distances 1: 0.1025 and 2: 0.1448, *P* = 0.001). Time affected the calf fecal community structure (*P* = 0.001, Fig. 2), with every day being different from each other (*P* = 0.001). In addition, dispersion among samples from the centroid of each day was significantly different when compared to each day (all *P* < 0.003), except for the dispersion between days 28 and 56, and 84 and 112. In summary, the microbiome composition was altered from 0 to 28 d, and there was less variability between animals at later time points.

The calf fecal bacterial community was mainly composed of the *Firmicutes* (41.50%) and *Bacteroidetes* (37.39%) phyla, followed by *Actinobacteria* (7.47%) (see Additional file 1: Fig. S1a). At the family taxonomical level, the calf fecal microbiota was mainly composed of *Ruminococcaceae* (15.29%), *Prevotellaceae* (15.04%), *Lachnospiraceae* (11.44%), and *Bacteroidaceae* (10.17%) (see Additional file 1: Fig. S1b). At the genus taxonomic level, the most abundant genera in the calf fecal microbiota were *Prevotella* (18.13%), *Bacteroides* (10.17%), and unclassified *Ruminococcaceae* (8.86%) (see Additional file 1: Fig. S1c).

**Fig. 3:**
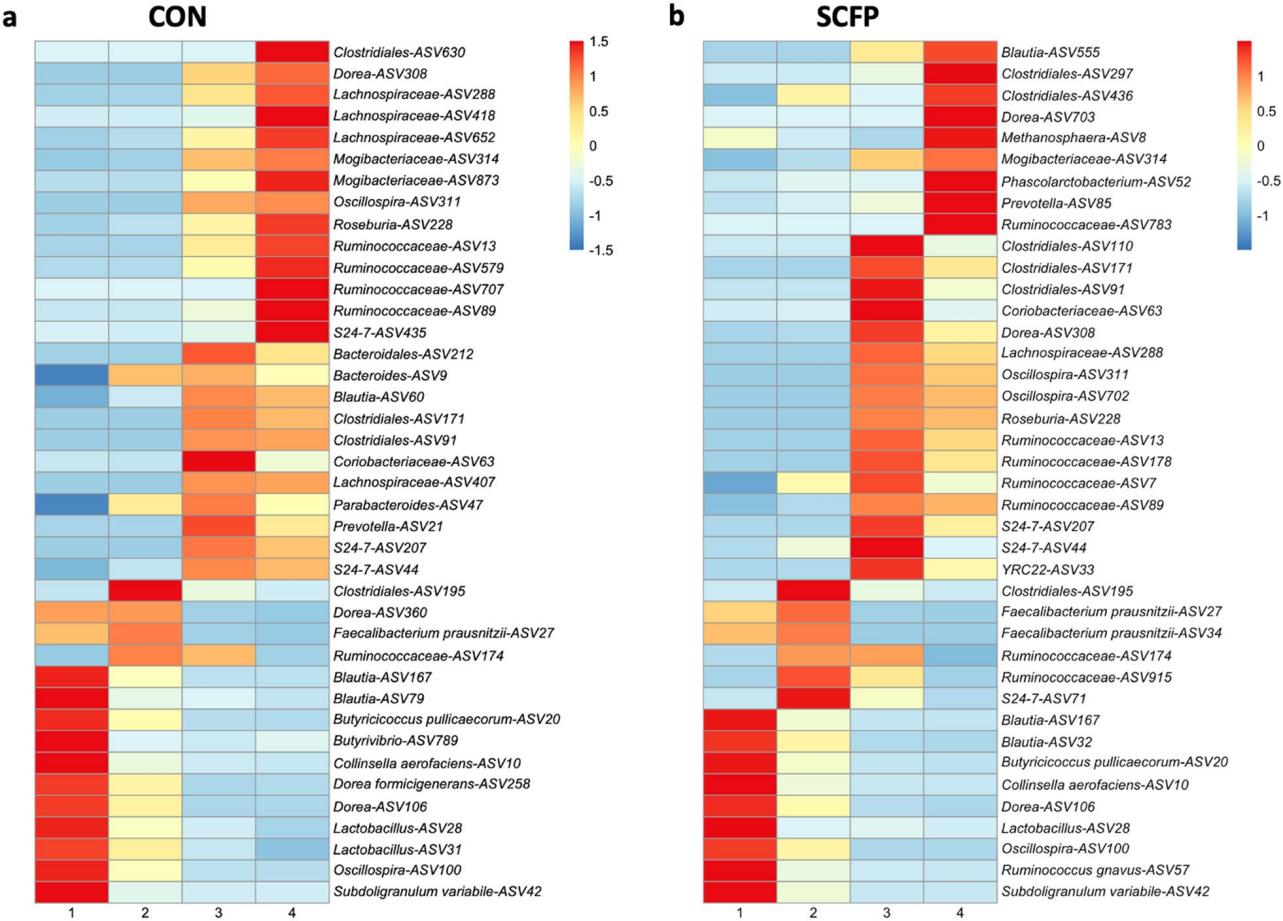
The 40 ASVs with the highest importance to the random forest regression model for each of the treatment groups: CON (**a**) and SCFP (**b**). The scale indicates the z-score, or the number of standard deviations away from the mean of all time points for each treatment group. Numbers in the x-axis represent the calf age in months: 1 = 28 d, 2 = 56 d, 3 = 84 d, and 4 = 112 d. The ASV number represent the different ASV assigned to the same taxonomical category but indicates a different sequence variant

When comparing the calves fecal community composition as determined by 16S rRNA sequencing between SCFP vs CON from day 28 to 112 using a random forest (RF) regression, the ASV table used as the input explained 89.94% of the variance with 0.12 mean squared residuals in the CON group, and 93% of the variance explained with 0.09 mean square residuals in the SCFP. From the two regression models, 40 ASVs were selected and identified in each treatment group to have an importance in the accuracy of the model (approximate model error: CON: 0.118 and SCFP: 0.089, see Additional file 1: Fig. S2). Minimal improvement in the predictive performance was observed when selected taxa beyond the top 40 for each group (see Additional file 1: Fig. S2). The taxa with the highest mean decrease accuracy (> 0.1) in the CON group, which indicate the importance of the ASV in the accuracy of the model, were ASVs assigned to *Collinsella aerofaciens, Oscillospira* and *Dorea*, while two unclassified *Ruminococcaceae, Clostridiales,* and *Subdoligranulum variabile* had the highest mean decrease accuracy in the SCFP group. The 40 most influential ASVs for the CON and SCFP groups belonged to 20 genera from which, 4 genera were not shared between the two groups (see Additional file 1: Table S1). From these 40 ASVs (see Additional file 1: Fig. S2), more than four belonged to *Ruminococcaceae, Clostridiales, Dorea* and *Lachnospiraceae* (CON group*), Ruminococcaceae* and *Clostridiales* (SCFP group). Therefore, the implementation of RF regression analysis allowed the identification of influential taxa present in the fecal microbiome between CON and SCFP group and how their abundance changed through time. When considering the abundance of the 40 most influential ASV in the RF model for each group from day 28 to 112, the CON group had the most ASVs (n = 14) with high z-score (> 1), or the number of standard deviations away from the experiment-wide mean, for the fourth month of age (d 112), while the SCFP group had the most ASVs (n = 17) with high abundance during the third month of age. Many predictive ASVs reached their highest abundance during the same time period in both treatment groups; however, some the timing of when the 40 most influential ASVs were most abundant (z-score) was different between the two groups (Fig. 3). On the first month (28 d), *Butyrivibrio, Dorea formicigenerans* and *Lactobacillus* were predictive of the CON group and not the SCFP group*,* while *Ruminococcus gnavus* was uniquely predictive of the SCRP group. On the second month, one unclassified *Clostridiales* ASV seemed to have high predictive power (z-score > 1, Fig. 3) regardless of the treatment, as well as *Faecalibacterium prausnitzii* and unclassified *Ruminococcaceae* to a lesser extent. On the third month, we observed ASVs from 6 genera to be uniquely predictive of the CON treatment, but the SCFP group was uniquely predicted by *Clostridiales*, *Dorea*, *Oscillospira*, *Roseburia*, and unclassified *Ruminococcaceae*. Lastly on the fourth month, ASVs classified as unclassified *Ruminococcaceae,* unclassified *Lachnospiraceae*, and unclassified *Mogibacteriaceae* were more predictive of the CON group, while the SCFP group contained a more diverse predicted genera (Fig. 3). From the 40 representative ASVs, 22 were shared between the two groups (see Additional file 1: Fig. S3). Interestingly, 6 of these shared ASVs (*Dorea-*ASV308, *Lachnospiraceae-*ASV288, *Oscillospira*-ASV311, *Roseburia*-ASV228, *Ruminococcaceae-*ASV89 and *Ruminoccocaceae*-ASV13) reached their highest abundance in the third month on the SCFP animals but on the fourth month in the CON group (see Additional file 1: Fig. S4). In summary, supplementation of SCFP affected the abundance of specific fecal microbiota members, and selected them to peak in abundance earlier in time.

**Fig. 4:**
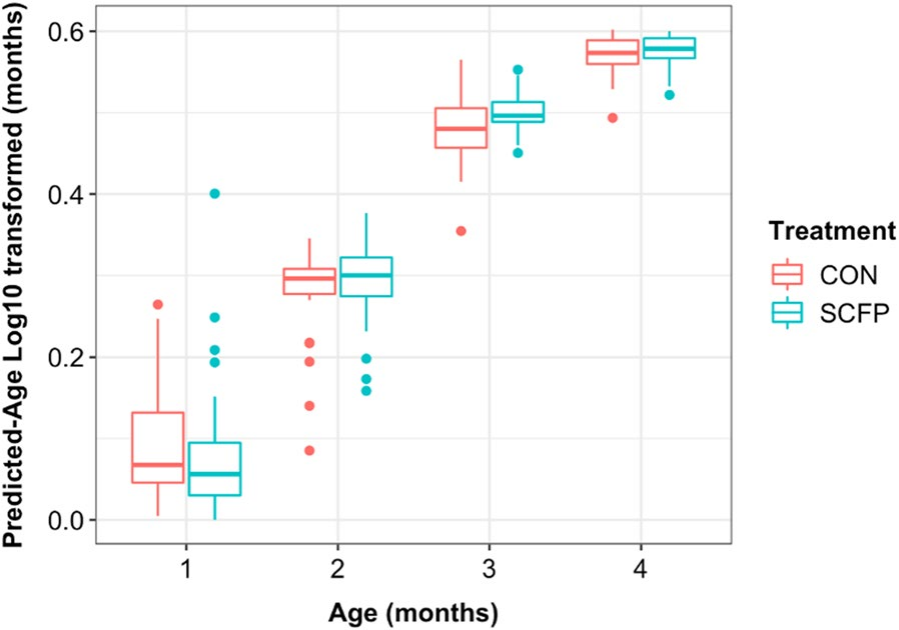
Correlation of animal age with the predicted-age log_10_ transformed using the 40-ASV RF model between CON and SCFP group across months of age. Numbers in the x-axis represent the four months of age (1 = 28 d, 2 = 56 d, 3 = 84 d, and 4 = 112 d). Samples from the CON treatment represent the age-discriminatory model training set and samples from the SCFP represent the model applied (testing set)

To determine the fecal microbial succession or maturation across CON and SCFP groups during its first 4 month of age, a RF-regression model was creating using the ASV table as input and samples from the CON (training set). This allowed the creation of model that identified bacterial taxa that discriminate the calves’ fecal microbiome maturation from day 28 to 112 of the study. From the model, 40 ASVs were selected by tenfold-cross-validation with an approximate error of 0.118 and 89.94% of the variance explained. To test the model, the 40 age-discriminatory taxa model was applied to the SCFP samples (testing set). A positive correlation was observed between the RF predicted age and the actual age (in months) (df = 155, r = 0.97, *P* = 4.01e−100, 95% CI: 0.96–0.98, Fig. 4). Interestingly, there was no significant difference in the microbiome predicted age of animals between the two treatments (*P* > 0.05) but every month was well predicted, being significantly different from every other month (F 1, 53.00 = 1865.65, *P* < 0.001).

## Discussion

The goal of calf feeding system is to provide the animals the optimum nutrition to promote its growth, health and development. An important factor in maintaining the animal health it’s their gut microbiome composition [2–4]. Nonetheless, the utilization of antibiotics in the calf system to maintain the animals health can increase the development of antibiotic resistant bacteria stating the importance to find alternatives to the antibiotic use. In a companion study, calves were fed *Saccharomyces cerevisiae* fermentation products (SCFP) during their first 4 months of age [15] and demonstrated that supplementation of SCFP improved the ADG, BW, HW in dairy calves post-weaning and decreased the incidence of respiratory diseases. Therefore, this study highlights the changes on the dairy calves fecal microbiome composition and succession during the first 4 months of age and supplemented with SCFP. Our study clearly indicates that with time, the fecal microbiome of calves increases in both richness and evenness, from day 0 to day 112, as observed in other calves studies [16, 17]. Kim et al. [17], reported that an increase in fecal microbiome diversity was observed in dairy calves from 1 to 5 weeks of age; however, the composition and diversity of the fecal microbiome stopped increasing after week 6. In addition, Massot et al. [17] reported a sharp increase in bacterial richness the first month followed by a slower increase thereafter. Thus, it seems that increasing bacterial richness over time is a fairly generalizable occurrence. In addition, based on the beta diversity results, we observed that with time, inter-sample differences were reduced, as seen by decreased dispersion at d 56, 84, and 112 compared to d 0 and 28. Other studies have reported that the fecal microbiome in post-weaning calves are more similar to each other compared to pre-weaning calves [18].

In our study, while time-matched beta diversity of the fecal community was unchanged due to SCFP treatment, changes in taxa abundance dynamics were observed in microbial community succession. Forty ASVs were identified as age discriminatory because they could be used to successfully predict animal age based on their abundance from day 28 to 112. From the 40 age discriminatory taxa, 22 ASVs were shared between the two groups; six of them (*Dorea-*ASV308, *Lachnospiraceae-*ASV288, *Oscillospira*-ASV311, *Roseburia*-ASV228, *Ruminococcaceae-*ASV89 and *Ruminoccocaceae*-ASV13) reached their maximum z-score at a different age between the two groups: at four months in the CON group, and at three months in the SCFP group. The use of “age-discriminatory taxa” was used to compare these two groups of animals because the bacterial community was under continual change over time, and time-matched comparison is unable to capture the rate of change of the community. This analytical approach was first attempted in human studies to determine the change in community succession in two groups of children with different growth phenotypes [19]. Thus, taken together, this suggests the possibility of utilizing the “age-discriminatory taxa” in the fecal microbiome as an indicator of animal health. In addition, it also highlights that the supplementation of SCFP could affect the abundance of some taxa to reach their high abundance earlier in time (animal age) compared to the CON group. Nonetheless, more research is needed to test the impact of the abundance of “age-discriminatory taxa” on animal growth and health.

In addition, during the first 4 months of age, the fecal microbiota was mainly composed of the *Firmicutes, Bacteroidetes* and *Actinobacteria* phyla, which have been previously identified as common members of neonatal calves [4]. At a genus level, *Prevotella, Bacteroides* and unclassified *Ruminococcaceae* were the most abundant regardless of the treatment and these have been previously identified as members of calves fecal microbiome [20, 21]. Previous studies [13] suggest that beneficial effects of SCFP on the rumen microbiome are mediated by increasing the relative abundance of several members of *Bacteroidetes* phylum, such as *Prevotella*, and some members of *Ruminococcaceae* and *Lachnospiraceae* families including genera *Dorea, Blautia* and *Roseburia*.

A total of 40 ASVs in each treatment were identified as the most predictive of animal age. In the CON group, ASVs identified as *Collinsella aerofaciens, Oscillospira,* and *Dorea* were the most influential for discriminating animal physiological age through 4 months of life. In the SCFP group, ASVs assigned to *Clostridiales, Subdoligranulum variabile,* and two unclassified *Ruminococcaceae* were the most influential for the model accuracy. In a different study, fecal samples from calves of four different breeds were collected and the fecal microbiome was used to predict gastrointestinal disease [22]. The authors identified that the presence of *Collinsella aerofaciens* was associated with healthy clinical outcome. In our study, *Collinsella aerofaciens* was the genera with the highest accuracy value for the CON RF model followed by *Oscillospira*. In a study comparing healthy and hemorrhagic diarrheic calves, calves with diarrhea presented a significant decrease in the relative abundance of *Oscillospira* found fecal swabs [23]. In the case of the SCFP, two ASVs assigned to the family *Ruminococcaceae* were important of the model accuracy. Similar results were observed in which the abundance of *Ruminococcaceae* in rectal samples collected at slaughter on day 56 were increased in SCFP compared to CON animals [24]. The authors concluded that the high abundance of *Ruminococcaceae* could be liked with cellulose degradation in the large intestine. In addition, it has been shown that SCFP supplementation can increase VFA production [25]. Interestingly, in our study, the species *Subdoligranulum variabile*, a butyrate-producing bacterium [26], was identified by the SCFP RF model as one of the predictive bacteria in the model. This supports the evidence that SCFP supplementation influences the microbial community composition of the gastrointestinal tract. From these results is evident that the implementation of a random forest regression analysis provides the capacity to identify specific and important community members that can be identified as potential markers for each treatment.

In the current study, we found a difference in the fecal bacterial alpha and beta diversity between batch (1 and 2) and animal age. Dairy calves used in the study were transported to the university site in two different batches, the first batch was received in mid-Spring and the second batch in late-Summer. In previous studies, it has been shown that environmental temperature can cause heat stress that then affects the intestinal bacterial community [27, 28]. In addition, the difference between batches could be explained by the inherit variation that occurs between animals. This indicates that the fecal microbial community can be affected by multiple factors or by individual animal variation, and it is necessary to be careful when comparing results across studies.

## Conclusion

This study illustrated that age was the strongest driver of the fecal microbiome of calves specifically by increasing the richness and evenness of the bacterial community. The administration of SCFP increased the abundance of six ASVs at a younger animal age than in the control group. In addition, even though SCFP treatment did not have a major effect in the fecal microbiome community structure, there was a tendency for SCFP to increase microbial community evenness. Our data also suggest that it is important to control for experimental design factors like batch and block to understand diet effects on the bacterial community. Lastly, more research is needed to fully determine if change in fecal microbiome succession rate is the mechanism by which calf health was improved.

## Methods

This study is a continuation of a companion paper and samples were analyzed based on the study design previously established [15]. Sixty Holstein bull calves, 5 ± 3 d of age (mean; ± standard deviation), were received in two separate batches (n = 30 each, 15 calves per treatment per batch) on May 24 and September 13, 2019, from a dairy farm 55 km from the University Dairy Farm (Purdue University Animal Science Research and Education Center), where they were placed in individual hutches with a fenced in area (length x width x height: 2.1 m × 1.1 m × 1.2 m, Calf-Tel), bedded with shavings and re-bedded as needed. On d 59 of the study, calves were weaned and moved from individual hutches to group hutches (6 group hutches, 4–5 calves in each). Calves were assigned to one of two treatments upon arrival at the Purdue Dairy Farm: CON or SCFP (n = 60: 30 calves per treatment). Animal diets were changed in 4 phases to meet the animal nutritional requirements: d 0 – 49 (24% crude protein:17% fat milk replacer (MR)), d 0 – 56 (calf starter), d 57 – 84 (grower 1) and d 85 – 112 (grower 2). No SCFP was added to the CON animal diets. SCFP calves received 1 g/d of SmartCare® (Diamond V, Cedar Rapids, IA) in milk replacer (MR), while NutriTek® (Diamond V, Cedar Rapids, IA) was added to the solid feed diets in a decreasing concentration (relative to dry matter): calf starter with 0.8% SCFP, grower 1 with 0.44% SCFP from d 57 to 84, and grower 2 with 0.275% (DM) SCFP from d 85 to 112. Calves for each batch (n = 30) were blocked by BW (low, intermediate and high BW, n = 10 calves in each block). Treatments (CON and SCFP) were randomly assigned to animals within each block, treatments were assigned evenly within each batch. The study ended on day 112. See Klopp et al., 2022 [15] for additional details on animal management and measurements.

Fecal samples were collected from each calf on 0, 28, 56, 84, and 112 d of the study via rectal stimulation, and immediately stored at 4 °C. Feces were collected using a 50 mL tube and placed in ice during the transportation to the lab. Once in the lab, the fecal samples were homogenized before DNA extraction. A representative fecal sample was placed in the DNA extraction plate and stored at −20 °C until DNA extraction using the MagAttract PowerMicrobiome DNA/RNA EP kit, following the manufacturer’s protocol, (Qiagen, Germantown, MD, USA). The V4 region of the 16S rRNA gene was amplified and an amplicon library pool was prepared following the Kozich et al. [29] protocol. The amplicons were sequenced with an Illumina MiSeq Sequencer (2 × 250 paired-end) at the Purdue Genomic Core Facility.

The raw sequencing data were analyzed using the Quantitative Insight Into Microbial Ecology (QIIME2) v.2020.2. DADA2 [30] was used to remove low quality sequences. The forward sequences were trimmed at 12 and 251, and the reverse sequences were trimmed at 12 and 233, attempting to maintain a quality score > q30 for more than 50% of the sequences at all positions. A total of 30,252,835 sequences were identified before the denoising step (DADA2), and after denoising, 24,394,047 sequences remained in the dataset. The taxonomy was assigned using the Greengenes 13_8, 515/806R classifier [31]. Then, the sequences were rarefied to 19,681 reads per samples to calculate the alpha and beta diversity of the community. Alpha-diversity was estimated with observed features as an indicator of richness and Pielou index [32, 33] as a measure of evenness. Beta-diversity, which measures the dissimilarity of microbial community structure between samples, was determined using weighted UniFrac [34] and plotted as principal coordinate analysis (PCoA) using R (v 4.1.2).

Alpha diversity data were analyzed as a completely randomized block design using the Mixed Procedure and GLM Procedure of SAS v.9.4 with repeated measures when applicable. The fixed effects included treatment (CON or SCFP), time point (0, 28, 56, 84, or 112 d), batch (1 or 2), block (1, 2, or 3 within each batch based on initial BW and STP), and the interactions between (1) treatment and time point, (2) treatment and batch, and (3) treatment and block. A repeated measures model was run with calf nested within treatment as the subject. All alpha diversity data was analyzed with calf as the experimental unit. We determined a *P*-value ≤ 0.05 to be significant, and a *P*-value > 0.05 and < 0.10 to be a tendency. For the analysis of the beta diversity, under the effect of treatments (SCFP and CON), batch (1 and 2), block (1, 2, and 3) and its interactions in the calf fecal beta diversity, a Permutational Multivariate Analysis of Variance Test (PERMANOVA; *P* ≤ 0.05) test was performed separately for each day (0, 28, 56, 84, and 112 d). For time effect and time and treatment interaction, a PERMANOVA test was performed with the calf (experimental unit) specified as the blocking factor using the argument “strata”. Pairwise comparison analysis (*P* ≤ 0.05) of the different time points was evaluated with the function pairwise.adonis2, in R (v 4.1.2) [35]. Additionally, a dispersion test was performed using the betadisper function from the vegan package, using weighted UniFrac distances as input data. To analyze the time effect, a repeated measure analysis was used, in which the average distance from each day and the average distance for each animal across the different timepoints was calculated, accounting for the subject within error. We then performed an ANOVA for repeated measures to analyze the average distance between each timepoint. To analyze the average dispersion between treatment, beta diversity outputs were divided by day. If the effect of time was significant, we performed a Tukey-Kramer post-hoc comparisons, using *P* ≤ 0.05 as statistical significance.

To identify and analyze the most representative taxa of both treatment groups and how the abundance of these taxa change through time, we used a random forest (RF) regression analysis from the randomForest package in R (v 4.1.2). We used the raw ASV table (7510 ASVs) as the input to build the RF model and the data was separated into training and testing set. The training set was used to identify most representative ASVs for each treatment based on a cross-validation method (‘rfcv’ function) that indicates how much accuracy the model loses if the ASV is not included and based on the model error [36]. The number of trees used in the RF model, were identified by the number of trees in which the lowest Mean Square Error (MSE) was observed. For the CON, the number of trees was 238, and this same number was applied in the SCFP RF regression model to maintain consistency in the iteration. After obtaining the most representative ASVs for the different time points (28, 56, 84, and 112 d), a heatmap was generated using the pheatmap package in R (v 4.1.2) to plot the changes in abundance calculated based on a z-score that indicates the distance between the raw data from the mean of the sample. Data on 0 d was not included in the analysis because experimental diets began after day 0.

Previous studies identified fecal bacterial members that are correlated with the individual age indicating that the gut microbiota goes through a normal maturation process (“microbiota age”) as the individual gets older [37]. Therefore, we created a model for microbiota age of the CON animals (training set) by using the RF to regress the most representative ASVs (obtained from the first RF regression model for the CON group) against the different timepoints (28, 56, 84, and 112 d). In this model, 128 trees were set based on the MSE. Then, the model was applied to the samples from the SCFP group (testing set). Lastly, a repeated measures correlation test was performed between the predicted RF-microbiota age and the actual age using the rmcorr [38] package in R (v 4.1.2), *P*-value ≤ 0.05 was set as a significant result. A linear regression mixed model (using the package afex) was applied to analyze the effect of time and time and treatment interaction in the RF predicted age. The different months, time, and treatment were included as the fixed factors. The random effect was specified as uncorrelated varying slopes and intercepts for each animal at each different month (d || subject ID). We checked assumptions of normality of the residuals and homogeneity of variance were checked using the afex package. The dependent variables were log-transformed when the assumptions were not met. All statistical analysis of the calf fecal microbiome involving was completed in R (v 4.1.2).

## Supplementary Information

**Additional file 1**. Supplementary Results. **Table S1.** Count table of the different ASVs assigned to the 40 most influential ASVs for each RF model (CON or SCF). **Figure S1.** Average relative abundance of the 15 most abundant phyla (a), family (b) and genera (c) present in the fecal microbiome of Holstein calves. * represents ASVs that were unclassified at the family level or higher. **Figure S2.** The 40 most influential ASVs determined by random forest model for CON (a) and SCFP (b) treatments. Mean decrease error (IncMSE) of each ASV is plotted in the x-axis. Color indicates the phyla for each ASV. Graph indicate the error model depending on the total of ASV (n.var) was used to determine the number of ASVs to include in the model. **Figure S3**. Shared ASVs between the CON (a) and SCFP (b) RF model. **Figure S4. **Heatmap representing the change in abundance of the 22 shared ASVs between CON (a) and SCFP (b).The scale indicates the z-score, or the number of standard deviations away from the mean of all time points for each treatment group. Numbers in the x-axis represent the sampling month: 1 = 28 d, 2 = 56 d, 3 = 84 d, and 4 = 112 d.

## Data Availability

Sequences were deposited in the NCBI sequence read archive (SRA) database under BioProject PRJNA699317, BioSamples SAMN17773447-SAMN17773722. Additional files used in data analysis for this study are available at https://github.com/EuniceCenteno/CalfFecalMicrobiome. for reference and reproducibility.

## Abbreviations

BRD: Bovine respiratory disease
SCFP: *Saccharomyces cerevisiae* fermentation products
CON: Control group
PCR: Polymerase chain reaction
ASVs: Amplicon sequence variants
QIIME: Quantitative Insight Into Microbial Ecology
PCoA: Principal coordinate analysis
PERMANOVA: Permutational multivariate analysis of variance test
RF: Random forest
BW: Body weight
STP: Serum total protein
d: Day
MSE: Mean square error
BW: Body weight
ADG: Average daily weight
HW: Hip width
