# Supplementary material for: Effects of feeding Saccharomyces cerevisiae fermentation postbiotic on the fecal microbial community of Holstein dairy calves
Source: Anim Microbiome. 2023 Feb 19;5:13. doi: 10.1186/s42523-023-00234-y (PMC9938967; doi:10.1186/s42523-023-00234-y)
Supplement: Supplementary file 1 — Additional file 1. Supplementary Results. Table S1. Count table of the different ASVs assigned to the 40 most influential ASVs for each RF model (CON or SCF). Figure S1. Average relative abundance of the 15 most abundant phyla (a), family (b) and genera (c) present in the fecal microbiome of Holstein calves. * represents ASVs that were unclassified at the family level or higher. Figure S2. The 40 most influential ASVs determined by random forest model for CON (a) and SCFP (b) treatments. Mean decrease error (IncMSE) of each ASV is plotted in the x-axis. Color indicates the phyla for each ASV. Graph indicate the error model depending on the total of ASV (n.var) was used to determine the number of ASVs to include in the model. Figure S3. Shared ASVs between the CON (a) and SCFP (b) RF model. Figure S4. Heatmap representing the change in abundance of the 22 shared ASVs between CON (a) and SCFP (b).The scale indicates the z-score, or the number of standard deviations away from the mean of all time points for each treatment group. Numbers in the x-axis represent the sampling month: 1 = 28 d, 2 = 56 d, 3 = 84 d, and 4 = 112 d. [file 42523_2023_234_MOESM1_ESM.pdf]

ADDITIONAL FILE 1

Effects of feeding *Saccharomyces cerevisiae* fermentation postbiotic on the fecal microbial community of Holstein dairy calves.

Ruth Eunice Centeno-Martinez, Wenxuan Dong, Rebecca N. Klopp, Ilkyu Yoon, Jacquelyn P. Boerman, Timothy A. Johnson

Additional file 1: Supplementary Results.

Table S1. Count table of the different ASVs assigned to the 40 most influential ASVs for each RF model (CON or SCF).
Figure S1. Average relative abundance of the 15 most abundant phyla (**a**), family (**b**) and genera (**c**) present in the fecal microbiome of Holstein calves. * represents ASVs that were unclassified at the family level or higher.
Figure S2. The 40 most influential ASVs determined by random forest model for CON (a) and SCFP (b) treatments. Mean decrease error (IncMSE) of each ASV is plotted in the x-axis. Color indicates the phyla for each ASV. Graph indicate the error model depending on the total of ASV (n.var) was used to determine the number of ASVs to include in the model.
Figure S3: Shared ASVs between the CON (a) and SCFP (b) RF model.
Figure S4. Heatmap representing the change in abundance of the 22 shared ASVs between CON (a) and SCFP (b).The scale indicates the z-score, or the number of standard deviations away from the mean of all time points for each treatment group. Numbers in the x-axis represent the sampling month: 1 = 28 d, 2 = 56 d, 3 = 84 d, and 4 = 112 d.

Table S1. Count table of the different ASVs assigned to the 40 influential ASV for each RF model (CON or SCF).

**CON**	**ASVs**	**SCFP**	**ASVs**
Ruminococcaceae	5	*Ruminococcaceae*	7
Clostridiales	4	*Clostridiales*	6
Dorea	4	*Dorea*	3
Lachnospiraceae	4	*Lachnospiraceae*	1
Blautia	3	*Blautia*	3
S24-7	3	*S24-7*	3
Mogibacteriaceae	2	*Mogibacteriaceae*	1
Lactobacillus	2	*Lactobacillus*	1
Oscillospira	2	*Oscillospira*	3
Bacteroidales	1		
Bacteroides	1		
Butyricicoccus	1	*Butyricicoccus*	1
Butyrivibrio	1		
Collinsella	1	*Collinsella*	1
Coriobacteriaceae	1	*Coriobacteriaceae*	1
Faecalibacterium	1	*Faecalibacterium*	2
Parabacteroides	1		
Prevotella	1	*Prevotella*	1
Roseburia	1	*Roseburia*	1
Subdoligranulum	1	*Subdoligranulum*	1
		Methanosphaera	1
		Phascolarctobacterium	1
		YRC22	1
		Ruminococcus	1

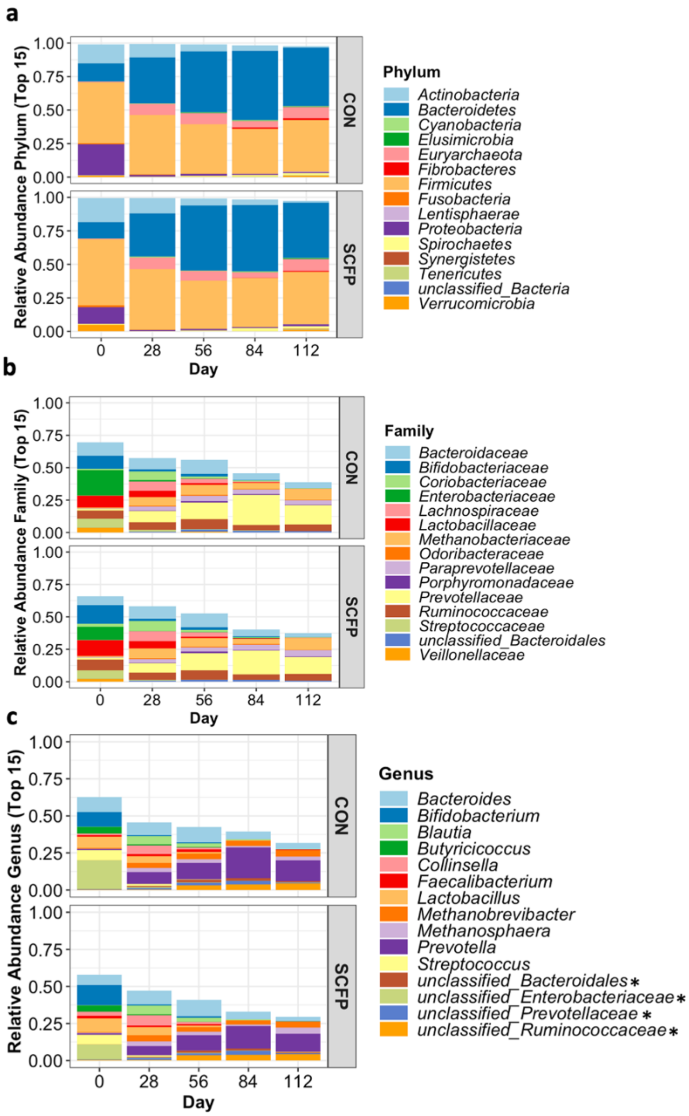

Figure S1. Average relative abundance of the 15 most abundant phyla (**a**), family (**b**) and genera (**c**) present in the fecal microbiome of Holstein calves. * represents ASVs that were unclassified at the family level or higher.

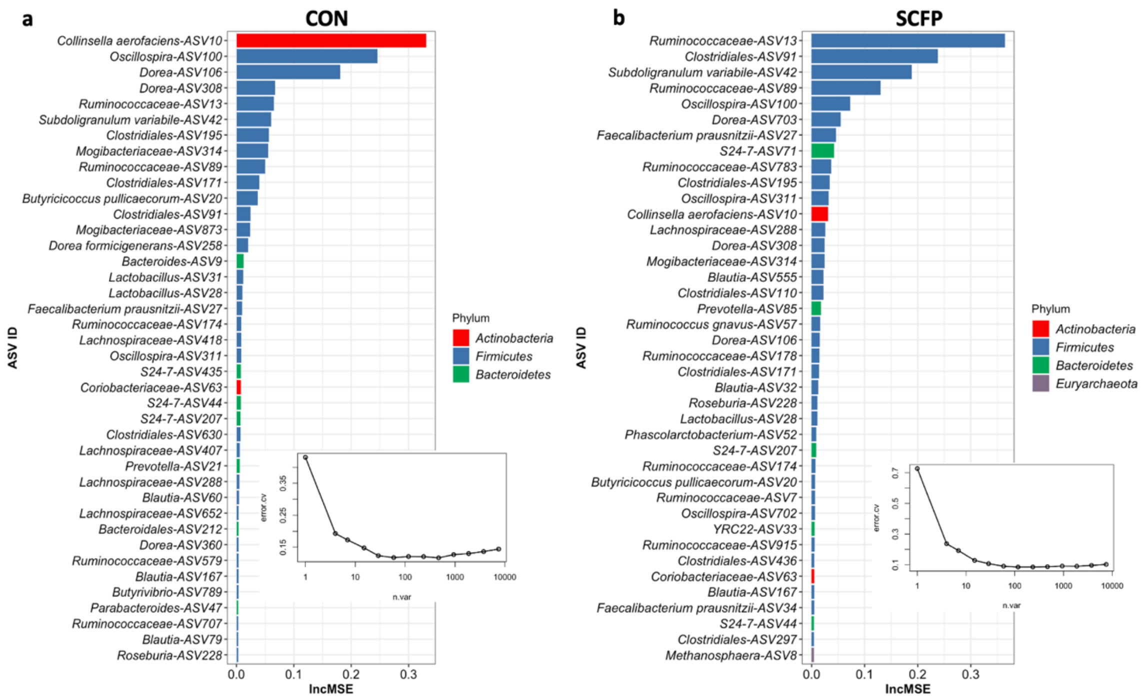

Figure S2. The 40 most influential ASVs determined by random forest model for CON (a) and SCFP (b) treatments. Mean decrease error (IncMSE) of each ASV is plotted in the x-axis. Color indicates the phyla for each ASV. Graph indicate the error model depending on the total of ASV (n.var) was used to determine the number of ASVs to include in the model.

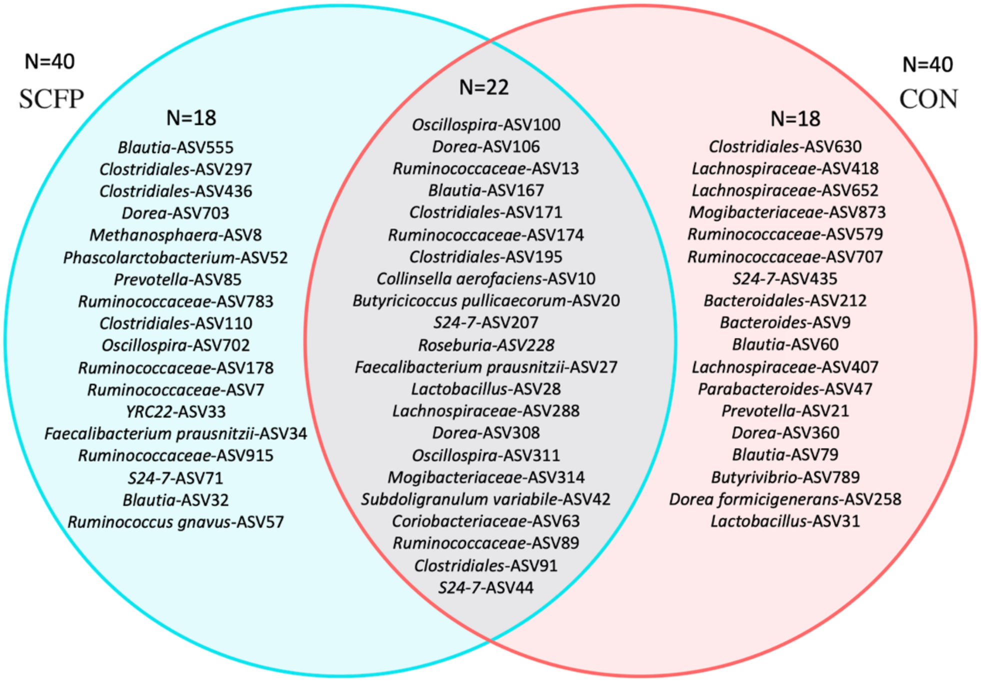

Figure S3: Shared ASV from the 40 ASV RF model between CON and SCFP model.

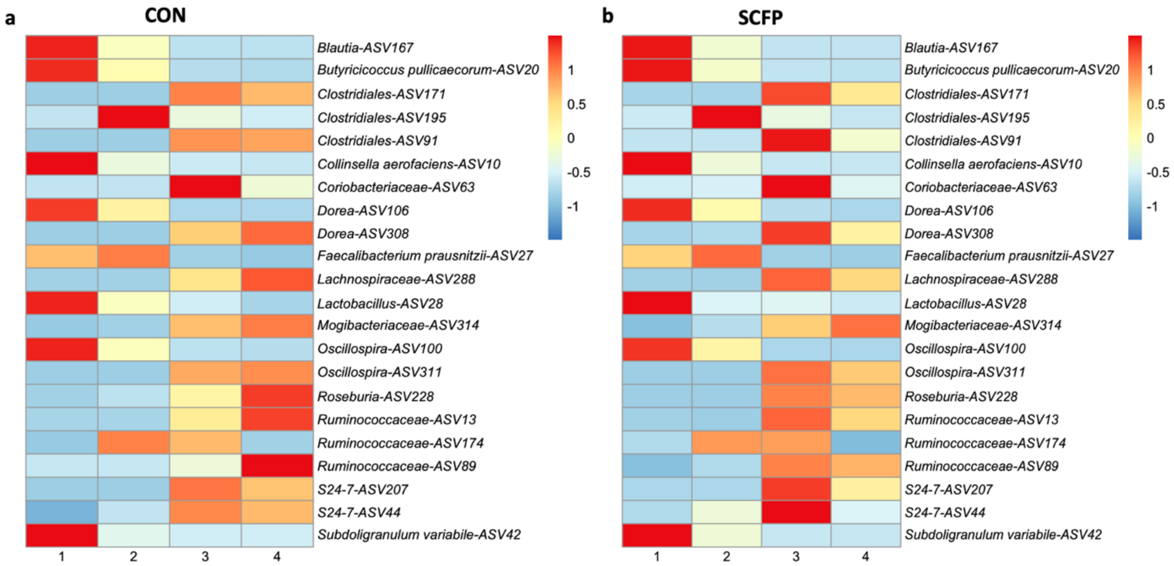

Figure S4. Heatmap representing the change in abundance of the 22 shared ASVs between CON (a) and SCFP (b). The scale indicates the z-score, or the number of standard deviations away from the mean of all time points for each treatment group. Numbers in the x-axis represent the days 1 = 28 d, 2 = 56 d, 3 = 84 d, and 4 = 112 d.
